# Disclosing transcriptomics network-based signatures of glioma heterogeneity using sparse methods

**DOI:** 10.1186/s13040-023-00341-1

**Published:** 2023-09-26

**Authors:** Sofia Martins, Roberta Coletti, Marta B. Lopes

**Affiliations:** 1 NOVA School of Science and Technology, NOVA University of Lisbon, Caparica, 2829-516 Portugal; 2 Center for Mathematics and Applications (NOVA Math), NOVA School of Science and Technology, Caparica, 2829-516 Portugal; 3 NOVA Laboratory for Computer Science and Informatics (NOVA LINCS), NOVA School of Science and Technology, Caparica, 2829-516 Portugal; 4 UNIDEMI, Department of Mechanical and Industrial Engineering, NOVA School of Science and Technology, Caparica, 2829-516 Portugal

**Keywords:** Glioma, Transcriptomics, Biomarkers, Sparse networks, Joint graphical lasso, Robust sparse K-means clustering

## Abstract

Gliomas are primary malignant brain tumors with poor survival and high resistance to available treatments. Improving the molecular understanding of glioma and disclosing novel biomarkers of tumor development and progression could help to find novel targeted therapies for this type of cancer. Public databases such as The Cancer Genome Atlas (TCGA) provide an invaluable source of molecular information on cancer tissues. Machine learning tools show promise in dealing with the high dimension of omics data and extracting relevant information from it. In this work, network inference and clustering methods, namely Joint Graphical lasso and Robust Sparse K-means Clustering, were applied to RNA-sequencing data from TCGA glioma patients to identify shared and distinct gene networks among different types of glioma (glioblastoma, astrocytoma, and oligodendroglioma) and disclose new patient groups and the relevant genes behind groups’ separation. The results obtained suggest that astrocytoma and oligodendroglioma have more similarities compared with glioblastoma, highlighting the molecular differences between glioblastoma and the others glioma subtypes. After a comprehensive literature search on the relevant genes pointed our from our analysis, we identified potential candidates for biomarkers of glioma. Further molecular validation of these genes is encouraged to understand their potential role in diagnosis and in the design of novel therapies.

**Supplementary Information:**

The online version contains supplementary material available at 10.1186/s13040-023-00341-1.

## Introduction

Gliomas are primary malignant brain tumors, accounting for 28% of all brain tumors and 80% of malignant ones [1]. The large heterogeneity characterizing glioma, at cellular and molecular levels, leads to distinct cancer types with different prognosis, among which glioblastoma (GBM) is the most aggressive one, with a median survival time of about 15 months [2]. Following the advances in molecular and cell technologies, relevant molecular information has been generated through transcriptomics and other ’omics profiling, enabling the definition of novel tumor classification and treatments [1]. Moreover, clinical-specific molecular biomarkers, namely, age and sex, have been pointed out by bioinformatic analysis disclosing several differences among clinical groups, e.g. at DNA methylation and gene expression levels [3–7]. However, a deeper molecular characterization of this cancer is necessary for an increased understanding of this type of tumors and the development of an effective personalized medicine.

The World Health Organization (WHO) classification of the Central Nervous System (CNS) tumors has been changing throughout the years regarding the classification of glioma. The classification of gliomas has evolved from histological-based [8] to increasingly based on molecular alterations combined to histological features [9, 10]. Glioma subtypes were divided into four main types: glioblastoma (GBM), astrocytoma, oligodendroglioma, and oligoastrocytoma, the latter presenting mixed histological nature, between astrocytoma and oligodendroglioma [8]. In 2016, molecular features were introduced to better define the glioma subtypes [9]. As a consequence, oligoastrocytoma diagnosis became strongly discouraged, and most of those cases could be reclassified as astrocytoma or oligodendroglioma. An updated version of the WHO CNS classification was introduced in 2021 [10], where oligoastrocytomas are no longer considered, and the glioma subtypes are classified mainly based on the sample’s molecular profiles.

The technological advances are responsible for the generation of large amounts of genetic information. With an increasing larger number of molecular features than samples, machine learning stands as a powerful tool to retrieve relevant information from these complex datasets. A relevant machine learning task to cope with ’omics data involves dimensionality reduction using, e.g., feature selection methods. Model regularization is a promising way to select features and improve model interpretability by adding constraints to the solutions. Methods like the least absolute shrinkage and selection operator (lasso) [11] and further versions (e.g., fused LASSO [12], adaptive LASSO  [13], and group LASSO [14]) and the elastic net [15] are examples of regularization methods. The lasso method was proposed for estimation in linear models by imposing a $L_1$-penalty on the cost function. Instead of focusing on subsets, this method defines a continuous shrinking operation capable of producing coefficients exactly equal to zero [11], making the solution sparse, and consequently, reducing the number of variables. The variables selected this way can be regarded as biomarker candidates for both diagnostics and therapeutic purposes [16].

Biological networks are one of the most studied types of networks used to decipher the molecular structures involved in disease development and progression [17]. Biological networks can be represented by graphs, where nodes are the biological entities and the edges the connections between them. Several studies acknowledge the role of networks in the study of cancer, beyond the selection of individual, potentially unrelated, molecular features. Several approaches have been proposed accounting for existing knowledge of gene interactions into classification models to identify network biomarkers and this way better understand the molecular mechanism behind cancer outcomes (e.g., [2, 17–20]).

Graphical lasso (glasso) [21] is a method for estimating undirected sparse graphs, enabling the identification of relevant subnetworks while discarding irrelevant links between the nodes, therefore inducing data dimension reduction. This method has shown successful disclosing gene interactions in 15 types of cancer [22], based on RNA-sequencing (RNA-seq) data. An extension of glasso, the joint graphical lasso (JGL), jointly estimates graphical models with observations from different classes [23], inducing sparsity and forcing similarity between these classes. JGL relies on the fact that it is expected that different but related classes (such as two different tumor subtypes) share some similarities. JGL determines the joint estimation of separated models, allowing for the exploration of similarities between multiple classes, maintaining the distinctive traits of each. This method stands promising for tackling cancer tumor heterogeneity in glioma, allowing for understanding how genes interact among different cancer glioma types.

Besides the relevance of identifying key network features in known cancer subtypes, the discovery of new patient groups from the increasingly available omics data is also encouraged. This approach aims to evaluate whether the information extracted from available cancer-type-specific molecular data correlates with the established clinical/molecular groups, or supports further cancer reclassification efforts. Finding groups of patients based on molecular information can be tackled by clustering methods. In the context of high-dimensional data, a shape-based method for sparse clustering, SPARCL, was proposed to allow clustering-based feature selection. The motivation behind this technique is to divide observations into a pre-specified number of groups, using only a subset of representative features. Robust K-means clustering (RSKC) is an extension of SPARCL that can handle outliers in the data. A pre-specified proportion of outliers is admitted by the method, which considers that the observations that are not so close to each cluster centers are not representative of any condition [24]. The ability to identify outliers is of high relevance in the context of gliomas, for which the classification has been evolving with successive alterations as far as more molecular information is considered in the definition of glioma subtypes.

The goal of this work is to identify, through sparse methods, transcriptomic network biomarkers of heterogeneity in gliomas. Although differences in glioma types have been investigated in the past [25–28], these studies are based on datasets collected or revised up to 2007. The successive release of updated glioma classification guidelines determined the change in some patients’ diagnoses, which could impact the glioma-type characterization. In this light, we are providing the first study exploring the consequence of the 2016 WHO glioma classification in biomarker discovery, aimed at either supporting this glioma classification or disclosing new patient groups.

In a first stage of the network-based methodology proposed, RNA-seq data from glioma patients were obtained from The Cancer Genome Atlas (TCGA), the largest repository of multiple omics data concerning cancer in humans [29]. The TCGA datasets were updated according to more recent glioma classifications. Through the application of JGL, we investigated network differences and similarities between glioma types. In the second stage of the analysis pipeline, the genes involved in the glioma-type specific networks inferred by JGL were used as input in the RSKC algorithm, to assess their ability to group patients into the known glioma subtypes. The potential to disclose new patient groups was also investigated. The gene networks inferred from each glioma subtype, and their most representative features, will potentially lead to a more comprehensive understanding of the molecular landscape of glioma, providing valuable insights to the definition of improved diagnosis, novel therapeutic targets, and ultimately contributing to patient life quality.

## Materials and methods

### Data

The RNA-seq data used were collected by the TCGA Research Network. We downloaded TCGA-GBM [30, 31] and TCGA-LGG [32] projects, which group the glioma patients according to 2007 WHO classification [33] into the two classes of GBM and Lower Grade Glioma (LGG), the latter comprising all astrocytoma, oligodendroglioma and oligoastrocytoma samples. To avoid considering obsolete glioma types, such as oligoastrocytoma, we updated the dataset to the 2016 WHO classification, by following the procedure explained in Mendonça et al. [34]. In practice, oligoastrocytoma samples were mainly reassigned to astrocytoma or oligodendroglioma, depending on the status of the IDH gene family and 1p/19q codeletion.

The final dataset comprises of 622 patients, divided as 264 astrocytoma, 220 oligodendroglioma and 138 GBM. As required from JGL, only normally distributed variables were considered in our dataset. To ensure this assumption, we selected the features having normal distribution in accordance with the Jarque-Bera test [35], leading to a total of 16338 genes. The LGG and GBM datasets were extracted using the GDCquery R function from **TCGAbiolinks** package [36, 37]. The TCGA datasets were already normalized with Transcripts Per Million (TPM) and upper quantile normalization. For the JGL method, nonparanormal normalization with huge.npn function from **huge** R package [38] was applied to lead the variables normally distributed [39]. For the RSKC method, z-score [40] was used.

### Joint graphical lasso

Let X be the data matrix, $n \times p$, where *n* and *p* denote the number of observations and the number of features, respectively. If all the features are independent and identically distributed, glasso algorithm estimates the precision matrix $\Theta$, which is the inverse covariance matrix. 0s in ${\Theta }$ correspond to pairs of features conditionally independent from each other, considering all other variables. These conditional relationships between variables correspond to an undirected graph, where nodes denote features and edges the relationships between pairs of features [21]. Let us consider *D* distinct but related datasets $Y^{(1)},..., Y^{(D)}$, *D* $\ge$ 2. $Y^{(d)}$ is an $n_d \times p$ matrix, where all *p* features are common to all the *D* classes. Features are independent and identically distributed within each class. The JGL [23] algorithm estimates the vector $\widehat{\Theta }=\left( \widehat{\Theta }^{(1)},..., \widehat{\Theta }^{(D)}\right)$, containing *D* precision matrices, each one defining the undirected network existing in the corresponding dataset. These networks are estimated jointly, by inducing sparsity and similarity across the different dataset through a penalty function. The authors of JGL method proposed two different penalty functions, the Group Graphical Lasso (GGL), leading to similar pattern of sparsity, and Fused Graphical Lasso (FGL), encouraging similarity between the edges. In this work, we selected FGL, which, beside providing generally better performances in various applications [23], was more suitable for the purposes of our work. FGL regulates the sparsity through the parameter $\lambda _1$ (which induces the same degree of sparsity in all the datasets), and encourages similarity among the *D* datasets by the parameter $\lambda _2$.

### Sparse clustering

K-means clustering divides the *n* observations into *K* clusters $C_1,...,C_K$, by minimizing the average distance between the observation constituting each cluster. Further extensions of this method defined outlier-robust clusters, which is able to identify outliers by considering an additional parameter $\alpha$. This pre-defined value represents the percentage of observations that are excluded from a given cluster in each step, as the one having the larger distance from the cluster center (outliers) [41]. This algorithm has been recently modified in order to introduce sparsity in the robust K-means clustering process, by means of a lasso-type penalty, regulated by the parameter *L*1 (the lower the parameter value, the more sparsity will be induced) [42].

### Clustering validation

A mathematical validation with random baseline of the RSKC results has been performed. For each case of study, we generated 1000 random datasets with the same dimension, and we applied RSKC, by fixing $L1=2$ and testing $K\in \{2,\ 3\}$. For each random dataset, the corresponding unsupervised clustering was evaluated by computing *silhouette* and *Calinski-Harabasz* scores.

### Analysis workflow

The analysis started by data preprocessing (dataset update and normalization), and visualization. The latter has been performed by using the Uniform Manifold Approximation and Projection (UMAP)[43], a nonlinear dimensionality reduction technique for data representation, widely used in multi-omics studies for sample visualization [44–46].

Three case studies were created to allow the comparison of different glioma subtypes, namely, ‘LGG *vs.* GBM’ (case A), ‘Astrocytoma *vs.* Oligodendroglioma’ (case B), and ‘Astrocytoma *vs.* Oligodendroglioma *vs.* GBM’ (case C).

The following step consisted of applying JGL to all three cases. JGL was applied using the R package JGL [23]. For cases A and B, we are assuming the existence of $D=2$ distinct datasets, while in case C the number of datasets is $D=3$. To detect the optimal choice for the JGL parameters, we tested several combinations, based on biological and practical considerations, as suggested by the JGL authors [23]. Given the high dimentionality of the considered starting datasets (16338 variables), a great level of sparsity was desired (high values of $\lambda _1$). Conversely, since the aim of this study was to highlight differences between glioma subtypes, we decided to not force similarity (low values of $\lambda _2$). Based on these assumptions, we tested $\lambda _1 \in \{0.90, 0.95, 0.97\}$ and $\lambda _2 \in \{0.001, 0.005, 0.01\}$. By comparing the results obtained for all the combinations of the tuning parameters, we detected $\lambda _1 = 0.95$ and $\lambda _2= 0.01$ as the most suitable combination to discuss the related estimated network, due to the reasonable number of selected variables (easy to discuss but large enough to be biologically meaningful). In the Results and discussion section we focus on this outcome, by performing clustering based on the corresponding variable selection. Indeed, as a way to validate the biological meaning of the inferred networks, RSKC was chosen to evaluate whether distinct patient groups would be obtained based on the features selected by JGL, in an unsupervised way. The corresponding outcome should disclose if the selected features were able to separate either known glioma subtypes or new patient groups. RSKC was performed using the R package **RSKC** [42], by testing different combinations of parameters. Specifically, the percentage of outliers has been defined as $\alpha = 0.1$, as suggested by the RSKC developers, while the regularization parameter *L*1 has been considered in $\{2,24\}$, in order to compare clustering results in a setting of strong or weak variable selection (conversely from JGL approach, low values of *L*1 lead to stronger regularization). To set the number of expected clusters *K*, we observed that the defined cases of study contain two glioma types in case A and B, and 3 glioma types in case C. However, in case C, astrocytoma and oligodendroglioma cases could be also considered as unique class (LGG), so we decided to test $K\in \{2,3\}$. For each parameter combination, the quality of the clusters obtained was evaluated using the simplified *silhouette* score [47] and the *Calinski-Harabasz* index [48], widely used clustering validity indices in omics studies and top performing indices across several real datasets [45, 49–52]. In the Results and discussion section, we will discuss only the outcomes obtained with the combination leading to the best scores, but the complete analysis is reported in Supplementary Table S4. Clustering validation with a random baseline has been also performed to assess the reliability of our results. For more details we refer to Supplementary Material.

The datasets and R code used for this study will be made available upon request.

## Results and discussion

**Fig. 1:**
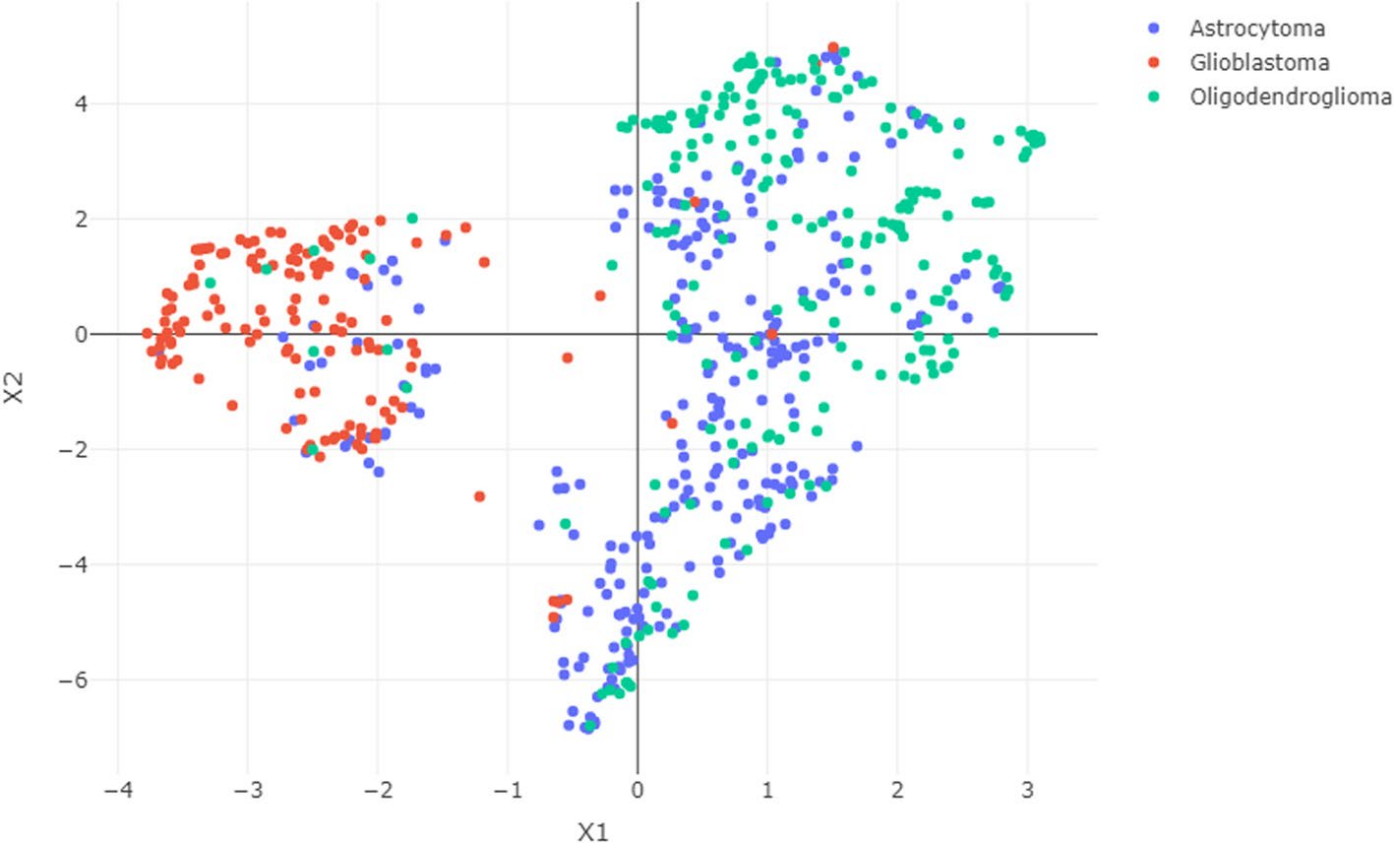
UMAP representation of the transcriptomics dataset. Labels are assigned based on the 2016 WHO glioma classification guidelines

A first visual inspection of glioma transcriptomics data was performed in a 2-dimensional space obtained via UMAP, in order to capture potential preexisting patterns. In this representation (Fig. 1), we can distinguish 2 well-separated groups, with LGG types (astrocytoma and oligodendroglioma) appearing closer compared to GBM cases. This outcome is in agreement with previous literature reports, which highlights some similarities among LGG types which affect tumor evolution and lead to better overall survival compared to GBM patients [53–55]. This preliminary result supports the need for further disclosing the molecular similarities and uniqueness governing glioma development and progression.

### Network inference

**Fig. 2:**
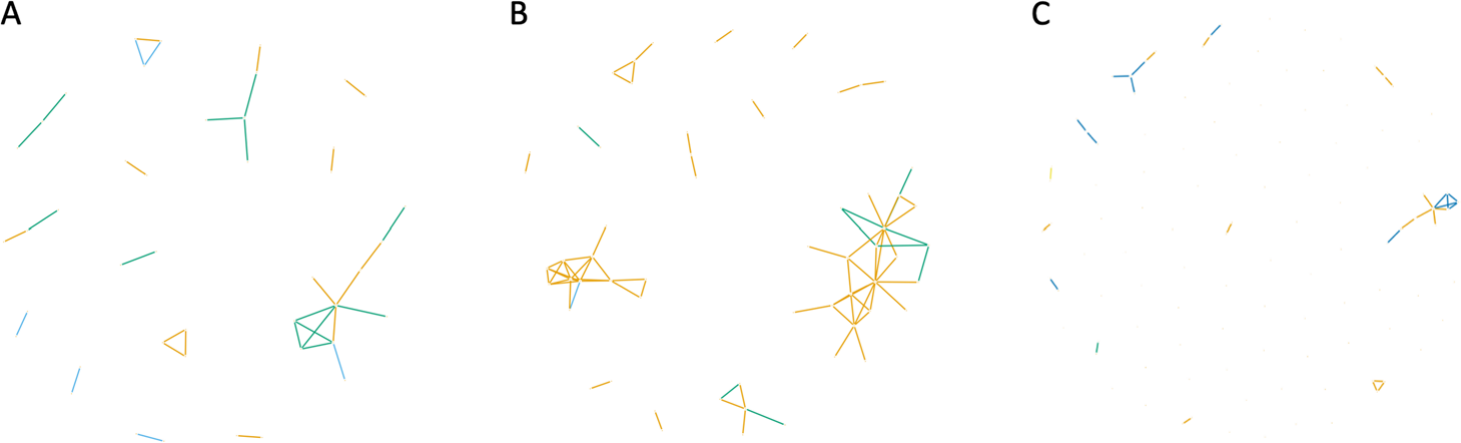
JGL networks with $\lambda _1 = 0.95$ and $\lambda _2 = 0.01$, for three cases of study: (A) LGG vs GBM, (B) astrocytoma vs oligodendroglioma, and (C) astrocytoma vs oligodendroglioma vs GBM. When considering two classes (A and B), shared edges are colored in orange, while exclusive edges related to the first and second class are green and blue, respectively. When considering three classes (C), shared edges across the three types are highlighted in orange, shared edges between astrocytoma and oligodendroglioma are in blue, shared edges between astrocytoma and GBM are in green, while exclusive GBM edges appear in yellow

Figure 2 illustrates a single network for each case study obtained through JGL with $\lambda _1=0.95$ and $\lambda _2=0.01$. Each variable is represented by a node (gene), while edges represent relations between nodes. The common edges, as well as the edges exclusive to each class, are highlighted with different colors.

For the ‘LGG *vs.* GBM’ case (Fig. 2A) the algorithm selects a total of 43 variables. There are some common edges (*n *= 14), but most of the estimated connections are exclusive for LGG (*n *= 14) and GBM (*n* = 6). A large subnetwork can be observed on the right hand side, containing 10 genes and both exclusive (green and blue) and shared connections between LGG and GBM (yellow). The genes included in this subnetwork are *TMEM125*, *ERMN*, *GJB1*, *CARNS1*, *KLK6*, *MAG*, *MOG*, *MBP*, *MOBP* and *CNDP1*. Half of them are involved in relations which are common to the two classes, while *CNDP1* is exclusive to GBM, and *TMEM125*, *ERMN*, *GJB1*, and *CARNS1* are nodes in the LGG network.

The network obtained for the ‘Astrocytoma *vs.* Oligodendroglioma’ case (Fig. 2B) is composed by 61 variables and shows many common edges between the two datasets (yellow, *n *= 65). There are only a few edges exclusive to one of the two subtypes, namely *n *= 9 for astrocytoma (green) and *n* = 1 for oligodendroglioma (blue). The network highlights the presence of a large subnetwork on the right hand side, represented by genes *BUB1B*, *CENPF*, *TPX2*, *AURKB*, *BIRC5*, *BUB1*, *CKAP2L*, *FAM64A*, *GTSE1*, *HJURP*, *KIFC1*, *MKI67*, *NCAPG*, *NCAPH*, *NUSAP1*, *PBK*, *TOP2A*, *TROAP*, *TTK* and *UBE2C*. All these genes are involved in relations described in both datasets, except for *BUB1B*, *CENPF*, *TPX2*, which are exclusive to astrocytoma.

The ‘Astrocytoma *vs.* Oligodendroglioma *vs.* GBM’ case (Fig. 2C) results in the most comprehensive network. It comprises 30 edges. Most of them are shared by astrocytoma and oligodendroglioma (*n *= 13, blue), and by all three subtypes (*n *= 15, orange). Only one edge is shared between GBM and astrocytoma (green), as well one edge has been estimated only in GBM dataset (yellow). There are no exclusive edges of astrocytoma or oligodendroglioma, and shared edges between oligodendroglioma and GBM. A subnetwork of shared edges can be observed, composed by the following genes: *TMEM125*, *ERMN*, *GJB1*, *MOG*, *CARNS1*, *KLK6*, *MAG*, *MBP*, and *MOBP*.

For each case, the 5 genes with the highest number of connections were selected. These genes, referred to as *hubs*, are listed in Table 1, where the number of connections is reported (in brackets).

**Table 1:** Hub genes selected by JGL for the three cases

LGG *vs.* GBM		Astrocytoma *vs.* Oligodendrogioma		Astrocytoma *vs.* Oligodendrogioma *vs.* GBM		
Hubs$_{LGG}$	Hubs$_{GBM}$	Hubs$_{Astro}$	Hubs$_{Oligo}$	Hubs$_{Astro}$	Hubs$_{Oligo}$	Hubs$_{GBM}$
*MAG*(6)	*MAG*(3)	*KIFC1*(10)	*KIFC1*(10)	*MAG*(6)	*MAG*(6)	*MAG*(4)
*GJB1*(3)	*ANXA2P1*(2)	*TOP2A*(10)	*MAG*(7)	*GJB1*(3)	*GJB1*(3)	*C1QA*(2)
*KLK6*(3)	*ANXA2P2*(2)	*TMEM125*(7)	*TMEM125*(7)	*KLK6*(3)	*KLK6*(3)	*C1QB*(2)
*TMEM125*(3)	*ANXA2*(2)	*MAG*(6)	*TOP2A*(7)	*TMEM125*(3)	*TMEM125*(3)	*C1QC*(2)
*TOP2A*(3)	*C1QA*(2)	*UBE2C*(6)	*UBE2C*(6)	*TOP2A*(3)	*TOP2A*(3)	*DOCK2*(2)

*LGG* lower-grade glioma, *GBM* glioblastoma

**Fig. 3:**
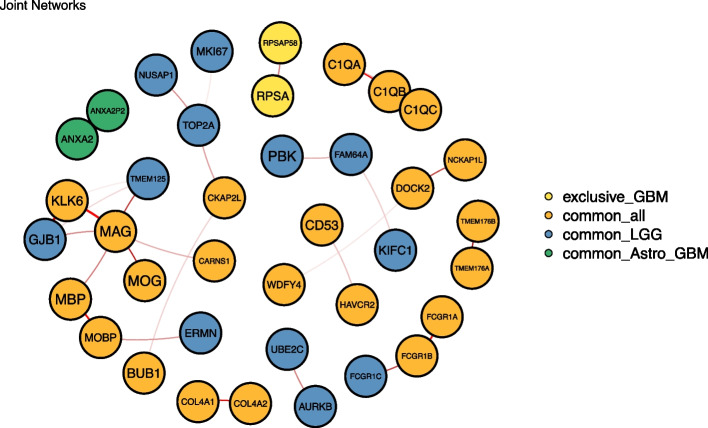
Case C joint networks representation with gene names. Colors are maintained as the ones provided by the JGL representation. Yellow nodes represent genes that only appear in the GBM network; orange nodes are genes shared across the three types; blue nodes are common between both LGG types (astrocytoma and oligodendroglioma); green nodes are common between astrocytoma and GBM

To further explore the estimated joint networks we focused on case C, i.e., the one considering the three glioma types. Figure 3 shows the same joint networks as in Fig. 2C, but emphasizing the nodes constituting the graph, instead of the edges. For consistency, nodes in orange represent genes that have been commonly selected by the three glioma types, while blue and green nodes are shared between LGG (astrocytoma and oligodendroglioma), and astrocytoma-GBM types, respectively. Yellow nodes are exclusive to GBM. In this representation, the relation between *RPSA* and *RPSAP58* appears as potentially relevant for GBM. On the other hand, two subnetworks related to LGG are detected, i.e., the one constituted by *PBK*, *FAM64A* and *KIFC1*, and the one involving *UBE2C* and *AURKB*. While the role of *RPSA* in GBM has not yet been investigated, the LGG genes we detected are all known in the context of glioma [56–59]. Interestingly, it has been recently discovered a combined effect of *UBE2C* and *AURKB* genes on glioma histology [60], which is the basis of the 2016 WHO classification. More details about the specific-gene role are provided in Supplementary Materials, Section 0.2.

### Clustering

RSKC was applied to the subsets of genes selected through JGL in each case. The rationale of this analysis was to evaluate whether the selected features would identify the known glioma subtypes or disclose new groups according to the RNA-seq data. Table 2 shows the best results obtained by RSKC for each of the combinations of parameters tested (*K* and *L*1). For all combination of parameters, the best performance was obtained for $L1=2$, hence the corresponding parameter value is not reported in Table 2 (complete results in Table S4). The quality of results was evaluated by the *silhouette* and the *Calinski-Harabasz* scores, which have been calculated by considering all cases, including the ones that the method identified as outliers. Larger values of the *silhouette* were obtained when removing the outliers from the dataset, as expected (Supplementary Table S4). For all the cases of study, the *silhouette* scores were higher than 0.5, indicating the good quality of the identified clusters [61]. Overall, the two indexes are in agreement, with the only exception of case A, where the *silhouette* indicates a better cluster with $K=2$, while *Calinski-Harabasz* is higher for $K=3$. This result is reasonable, since the starting dataset in case A was based on the variables selected by considering 2 classes, but the LGG class includes two distinct glioma types. In case B, the scores obtained for $K=2$ and $K=3$ are comparable, though both indicate a slightly better cluster division with $K=3$. Interestingly, in case C the better cluster division is the one provided by $K=2$, even if we are considering the 3 glioma types, separately.

**Table 2:** Summarized results of RSKC applied to the three cases considering the genes selected by JGL ($\lambda _1$= 0.095 and$\lambda _2$= 0.01), for$\alpha$= 0.1 and *L1 *= 2. The clusters were evaluated by Silhouette and Calinski-Harabasz scores. ARI was computed to quantify the agreement between clusters and the glioma types, considering the most relevant parameters combination

Case	K	*Silhouette*	*Calinski-Harabasz*	*ARI*
(A) LGG *vs.* GBM	2	0.70	640.95	0.54
	3	0.62	786.37	–
(B) Astrocytoma *vs.* Oligodendroglioma	2	0.58	513.29	0.15
	3	0.59	526.92	–
(C) Astrocytoma *vs.* Oligodendroglioma *vs.* GBM	2	0.73	843.31	0.49
	3	0.61	753.07	0.21

*LGG* Lower-Grade Glioma, *GBM* glioblastoma

To test the clustering robustness, we performed a validation with a random baseline. For all the cases of study, 1000 datasets composed of randomly selected subsets of variables of the same dimension of the variable sets selected by JGL were created. For each random dataset, RSKC was applied and the clustering performances were evaluated by computing the *silhouette* and *Calinski-Harabasz* indexes. Table 3 shows the results of the best random clusters, as well as the average and the median values compared to the reference score, i.e., the one reported in Table 2. This approach allows the exploration of the complete transcriptomics datasets, providing a graphical representation of the overall score distributions (Figs. S1 and S2). Due to the fact that the *Calinski-Harabasz* index does not have a defined cut-off value, this representation also serves to evaluate the goodness of the corresponding indexes obtained in the different cases of study, by comparing them with the general distribution. In particular, in all cases, the computed *Calinski-Harabasz* scores appear as the highest values compared with all the 1000 random subsets from the complete dataset.

**Table 3:** Validation of RSKC through random baseline clustering. This table shows the scores corresponding to the best clustering result, the average and the mean value from the 1000 random datasets, comparing it with the reference scores

Case	K	Reference score		Random score (Best – Average – Median)	
		*Silhouette*	*Calinski-Harabasz*	*Silhouette*	*Calinski-Harabasz*
(A) LGG vs GBM	2	0.70	640.95	0.72 — 0.52 — 0.52	668.21 — 170.93 — 158.06
	3	0.62	746.37	0.59 — 0.33 — 0.34	247.07 — 66.21 — 60.47
(B) Astrocytoma vs Oligodendroglioma	2	0.58	513.29	0.74 — 0.45 — 0.46	264.50 — 57.91 — 46.17
	3	0.59	526.92	0.60 — 0.32 — 0.31	61.16 — 8.24 — 6.92
(C) Astrocytoma vs Oligodendroglioma vs GBM	2	0.73	843.31	0.73 — 0.51 — 0.52	497.87 — 153.68 — 142.14
	3	0.61	753.07	0.64 — 0.33 — 0.33	300.62 — 62.19 — 55.98

Cases A, B and C represent our cases of study, respectively, ‘LGG vs GBM’, ‘Astrocytoma vs Oligodendroglioma’, and ‘Astrocytoma vs Oligodendroglioma vs GBM’

*LGG* lower-grade glioma, *GBM* glioblastoma

To further investigate the configuration of the identified unsupervised clusters, we compared them with the pre-assigned diagnostic labels to verify if they are in agreement. Table 4 summarizes the result of this comparison. For cases A and B, $K=2$ is considered, while in case C we decided to show both outcomes obtained with $K=2$ (the best according to both the considered score) and $K=3$ (the actual classes we were taking into account).

In case A (LGG vs. GBM), the identified clusters support the defined glioma types, since cluster 1 and 2 are mainly composed by LGG and GBM cases, respectively. In case B, clustering places the majority of oligodendroglioma samples into cluster 2, while cluster 1 is mainly composed by astrocytoma samples. However, $40\%$ of astrocytoma cases were also assigned to cluster 2. In case C, if we consider $K=3$ there is not a clear distinction of the glioma types in the three clusters. Cluster 1 is mainly composed of LGG samples, and cluster 2 contains mostly GBM, while cluster 3 is a combination of all glioma types. However, by setting $K=2$ the obtained clustering reproduces the outcome of case A, with a clear distinction of LGG in cluster 1 and GBM in cluster 2.

**Table 4:** Cross-comparison between the clusters obtained by RSKC on the datasets composed by the genes selected through JGL (applied to the three cases of study) and the pre-assigned glioma types (according to 2016 WHO CNS classification)

	Case A		Case B		Case C (*K *= 3)			Case C (*K *= 2)	
Cluster	1	2	1	2	1	2	3	1	2
LGG (Astrocytoma)	425	59	156	108	192	27	45	60	424
LGG (Oligodendroglioma)			40	180	179	12	29		
GBM	18	120	-	-	7	92	39	127	11

*LGG* lower-grade glioma, *GBM* glioblastoma

To quantify how much the identified clusters are in agreement with the diagnostic labels, we computed the Average Rate Index (ARI) in each case of study, by considering the combination of parameters leading to the best results. ARI score provides a measure of how much the clusters agree or disagree with the known labels, by varying in a range of $[-1,1]$, where the extreme values mean complete disagreement or agreement, respectively, and 0 corresponds to the random assignments. The results (Table 2) are in line with the previous observations. The computed coefficients highlight an overall agreement in cases A and C (*K* = 2), while in cases B and C (*K* = 3) are associated to very low values.

Clustering has been also used to assess the biological information carried by the set of selected variables. To this aim, we compared the clusters obtained by considering the complete dataset with the one related to our cases of study, by fixing *L1* = 2. We observed that, for *K* = 2, the *silhouette* score computed by considering the complete dataset with was totally comparable with the one related to cases A and C . In all these cases, the values are higher than 0.7 (Table 2), indicating good performances of the clustering method. Table 5 compares the cluster assignments by considering the complete variable set vs case A and C. Most samples are systematically associated to the same group, meaning that no relevant information might be lost despite a considerable dimension reduction (from 16K to around 40 variables). For *K* = 3 (Table S4), the lower values of *silhouette* indicate that the three glioma type are not easily distinguished based on transcriptomics data. With our variable selection (case C,* K* = 3) we are able to slightly improve the quality of clustering (Table S4), but we cannot assess that we have a good clustering performance. We hypothesize this could depend on the labels assigned by following the 2016-WHO classification, which could not be properly explained by the transcriptomics layer. Indeed, while the ARI of case C (*K* = 3) is considerably low (ARI = 0.21, S4), the one computed for the clustering taking into account the complete set of variables was very close to the random assignment (ARI = 0.0055, Table S4), proving that our variable selection is defining the three glioma types, though these not represent the best clusters based on transcriptomics data.

**Table 5:** Comparison of the number of samples constituting the clusters obtained by considering the complete set of variables (rows) and the subset of variables in the case studies A and C (columns)

		Case A		Case C (*K *= 2)	
		C1	C2	C1	C2
Complete dataset	C1	390	38	387	41
	C2	53	141	59	135

These outcomes refer to the best clustering performances, obtained for *K* = 2 and *L1* = 2

### Potential biomarker discovery

Our analysis highlighted 27 interesting genes, which have been identified either as nodes in subnetworks or as hubs. Literature research revealed that 17 of these genes have been already investigated in the context of glioma, and they are recognized to be involved in many common processes. For instance, $41\%$ of them influence glioma cell proliferation and/or migration [59, 62–65], whereas $47\%$ resulted as differentially regulated in glioma [66–71]. Other genes, namely *C1QA*, *C1QB*, *C1QC*, *ANXA2*, *CENPF*, *NCAPH*, *ERMN*, and *MOBP* have been pointed out as relevant through bioinformatic analyses on glioma datasets [66, 72–75], while *CARNS1* and *DOCK2* have not yet been linked with adult glioma, but they are known to play role in cancer-related processes [76, 77]. More details about the specific processes in which these genes are involved are reported in the Supplementary Material, Section 0.2 [78–92]. These genes represent a possibility for biomarker discovery, but further biological evaluations are needed to assess their potential.

## Conclusions

This work aimed at finding potential biomarkers of glioma heterogeneity. The results obtained confirm that astrocytoma and oligodendroglioma are more similar to each other at a transcriptomics level compared to GBM. In particular, our estimated networks show many common relations between the two LGG subtypes, while GBM shares few edges. The K-means clustering also confirms this outcome, since the lowest *silhouette* and *Calinski-Harabasz* scores have been obtained in case B (comparing the two LGG subtypes). Overall, clustering results have been used as a validation of JGL variable selection. Indeed, both the considered scores confirmed good clustering performances, suggesting that a representative subset of genes might have been identified. Clustering outcomes also indicate that the expression of few genes can distinguish different glioma conditions and disclose new groups of patients based on transcriptomics data, since better performances were obtained with lower values of *L*1. Interestingly, in case C, which compares the three glioma types, the best clustering was obtained by considering only $K=2$ classes. This result highlights the difficulty to distinguish between astrocytoma and oligodendroglioma groups, and it is in agreement with the preliminary UMAP outcome. The investigation of the cluster composition highlights a general agreement between clustering results and pre-assigned diagnosis in distinguishing LGG and GBM. Despite this, the unsupervised clusters do not entirely reflect the patients’ glioma types. In particular, the two LGG cases are not coherently distributed into the two clusters in case B, which, compared to the other cases of study, provides worst RSKC performances, suggesting that the used diagnostic labels are not well described by transcriptomics. This assumption is also supported by the results obtained in the comparison between clustering from the complete dataset and the one related to our case C (*K* = 3). Indeed, despite our variable selection provides a slight improvement, this is not enough to obtain a good distinction of the three glioma types. For future studies, it would be interesting to compare the present results with the ones using an updated dataset according to the 2021 classification, to evaluate whether the latest classification yields better separation between known glioma types or if it might reveal new findings. Moreover, multinomial classification models based on transcriptomics data, possibly combined with relevant clinical data (e.g., sex and age) will enable assessing the concordance of the updated glioma classes with the groups here estimated in an unsupervised way, and further evaluating the features explaining the differences between the groups.

Although our study leads to a list of potentially interesting genes, further analysis is necessary to sustain the already performed literature search. Indeed, on one hand, the existence of previous studies about the role of the identified genes in glioma processes can be seen as a preliminary validation, supporting our findings and the great potentiality of this study. On the other hand, the genes that have not been yet described in the context of glioma might be regarded as candidates for experimental validation and therapy research. Biologically testing the most promising candidates will be the natural next step to validate their role in the genesis, development, and progression of glioma.

### Supplementary Information

**Additional file 1.** Supplementary Material.

## Data Availability

The methodology used to build the updated TCGA glioma dataset used in this study is described in [34].

## Abbreviations

TCGA: The Cancer Genome Atlas
RNA-seq: RNA-sequencing
LASSO: Least absolute shrinkage and selection operator
GBM: Glioblastoma
WHO: World Health Organization
CNS: Central Nervous System
LGG: Lower-grade glioma
glasso: Graphical lasso
JGL: Joint Graphical Lasso
FGL: Fused graphical lasso
SPARCL: Shape-based clustering
RSKC: Robust k-means clustering
TPM: Transcripts per million
UMAP: Uniform manifold approximation and projection
ARI: Average Rate Index
