# Supplementary material for: Disclosing transcriptomics network-based signatures of glioma heterogeneity using sparse methods
Source: BioData Min. 2023 Sep 26;16:26. doi: 10.1186/s13040-023-00341-1 (PMC10523751; doi:10.1186/s13040-023-00341-1)
Supplement: Supplementary file 1 — Additional file 1. Supplementary Material. [file 13040_2023_341_MOESM1_ESM.zip › Martins_et_al_Supplementary.pdf]

# Supplementary Material

## 0.1 Joint Graphical lasso parameter testing

In this supplementary section, we show the results obtained for the tested parameter values for the JGL method. For each parameter combination, number of nodes and edges (belonging to one class, or shared) are reported. The parameter $\lambda_1$ regulates network sparsity (increasing its value, the total number of edges –and then the number of connected nodes – is reduced). The choice of $\lambda_2$ affects the similarity among the classes, which is illustrated by the number of shared edges (increasing $\lambda_2$ value, also the number of shared edges increases). For each class, the first 5 hub genes (highly connected nodes) are also showed. To assess the consistency of the JGL outcomes, we verified that, increasing the regularization parameters, we obtain a chain of subsets of nodes. For instance (Table S1), let $S_1$ be the set of 510 nodes obtained for $\lambda_1 = 0.9$ and $\lambda_2 = 0.001$, and $S_2$ the one with 71 nodes led by setting $\lambda_1 = 0.95$ and $\lambda_2 = 0.001$, then $S_2 \subset S_1$, and it occurs for all cases and parameter combinations.

Table S1: JGL parameter impact in case (A) LGG vs. GBM. LGG: Lower-Grade Glioma; GBM: glioblastoma.

| $\lambda_1$ | $\lambda_2$ | Connected Nodes | LGG Edges | GBM Edges | Shared Edges | LGG Subnetworks | GBM Subnetworks | 5 Hubs LGG | 5 Hubs GBM |
|---|---|---|---|---|---|---|---|---|---|
| 0.9 | 0.001 | 510 | 1737 | 551 | 217 | 47 | 67 | *NCAPG*(51)<br>*NUSAP1*(50)<br>*HJURP*(49)<br>*KIFC1*(48)<br>*TOP2A*(48) | *CD53*(27)<br>*SPI1*(25)<br>*VAV1*(25)<br>*LAPTM5*(24)<br>*NCKAP1L*(24) |
| 0.9 | 0.005 | 450 | 1532 | 507 | 260 | 41 | 65 | *NCAPG*(51)<br>*TOP2A*(47)<br>*HJURP*(46)<br>*KIFC1*(46)<br>*NUSAP1*(46) | *NCKAP1L*(28)<br>*CD53*(24)<br>*LAPTM5*(24)<br>*SPI1*(24)<br>*VAV1*(24) |
| 0.9 | 0.01 | 398 | 1316 | 452 | 299 | 40 | 58 | *KIFC1*(45)<br>*HJURP*(44)<br>*NCAPG*(44)<br>*TOP2A*(44)<br>*CDK1*(43) | *CD53*(25)<br>*NCKAP1L*(25)<br>*LAPTM5*(24)<br>*VAV1*(23)<br>*SPI1*(22) |
| 0.95 | 0.001 | 71 | 63 | 22 | 10 | 12 | 14 | *KIFC1*(9)<br>*TOP2A*(8)<br>*MAG*(6)<br>*TMEM125*(6)<br>*FAM64A*(5) | *ANXA2P1*(2)<br>*ANXA2P2*(2)<br>*ANXA2*(2)<br>*C1QA*(2)<br>*C1QB*(2) |
| 0.95 | 0.005 | 51 | 42 | 20 | 12 | 11 | 13 | *MAG*(6)<br>*TMEM125*(5)<br>*CARNS1*(4)<br>*FAM64A*(4)<br>*GJB1*(4) | *MAG*(3)<br>*ANXA2P1*(2)<br>*ANXA2P2*(2)<br>*ANXA2*(2)<br>*C1QA*(2) |
| 0.95 | 0.01 | 43 | 28 | 20 | 14 | 11 | 12 | *MAG*(6)<br>*GJB1*(3)<br>*KLK6*(3)<br>*TMEM125*(3)<br>*TOP2A*(3) | *MAG*(3)<br>*ANXA2P1*(2)<br>*ANXA2P2*(2)<br>*ANXA2*(2)<br>*C1QA*(2) |
| 0.97 | 0.001 | 11 | 4 | 3 | 1 | 3 | 3 | *KLK6*(2)<br>*C1QB*(1)<br>*C1QC*(1)<br>*GJB1*(1)<br>*MAG*(1) | *ANXA2P2*(1)<br>*ANXA2*(1)<br>*C1QB*(1)<br>*C1QC*(1)<br>*RPSAP58*(1) |
| 0.97 | 0.005 | 6 | 1 | 3 | 1 | 1 | 3 | *C1QB*(1)<br>C1QC(1) | *ANXA2P2*(1)<br>*ANXA2*(1)<br>*C1QB*(1)<br>*C1QC*(1)<br>*RPSAP58*(1) |
| 0.97 | 0.01 | 4 | 1 | 2 | 1 | 1 | 2 | *C1QB*(1)<br>*C1QC*(1) | *ANXA2P2*(1)<br>*ANXA2*(1)<br>*C1QB*(1)<br>*C1QC*(1) |

Table S2: JGL parameter impact in case (B) astrocytoma vs. oligodendroglioma.

| $\lambda_1$ | $\lambda_2$ | Connected Nodes | Astrocytoma Edges | Oligodendroglioma Edges | Shared Edges | Astrocytoma Subnetworks | Oligodendroglioma Subnetworks | 5 Hubs Astrocytoma | 5 Hubs Oligodendroglioma |
|---|---|---|---|---|---|---|---|---|---|
| 0.9 | 0.001 | 608 | 2334 | 2101 | 1236 | 55 | 51 | *NCAPG*(65)<br>*BUB1B*(62)<br>*TPX2*(60)<br>*FAM64A*(59)<br>*CDK1*(58) | *HJURP*(50)<br>*VSNL1*(48)<br>*MELK*(47)<br>*SLC17A7*(46)<br>*TOP2A*(46) |
| 0.9 | 0.005 | 547 | 2172 | 1975 | 1395 | 52 | 51 | *NCAPG*(62)<br>*BUB1B*(59)<br>*NUSAP1*(57)<br>*TPX2*(57)<br>*HJURP*(56) | *HJURP*(48)<br>*MELK*(48)<br>*TOP2A*(47)<br>*KIFC1*(45)<br>*NCAPG*(45) |
| 0.9 | 0.01 | 495 | 2033 | 1880 | 1548 | 51 | 50 | *NCAPG*(59)<br>*BUB1B*(54)<br>*KIFC1*(54)<br>*NUSAP1*(54)<br>*HJURP*(53) | *KIFC1*(48)<br>*MELK*(48)<br>*TOP2A*(48)<br>*HJURP*(47)<br>*NCAPG*(45) |
| 0.95 | 0.001 | 86 | 120 | 80 | 50 | 15 | 11 | *TOP2A*(15)<br>*NUSAP1*(12)<br>*KIFC1*(11)<br>*FAM64A*(10)<br>*TPX2*(9) | *KIFC1*(9)<br>*TOP2A*(9)<br>*MAG*(8)<br>*HJURP*(7)<br>*TMEM125*(7) |
| 0.95 | 0.005 | 74 | 88 | 71 | 59 | 15 | 12 | *TOP2A*(15)<br>*NUSAP1*(12)<br>*KIFC1*(11)<br>*FAM64A*(10)<br>*TPX2*(9) | *KIFC1*(9)<br>*TOP2A*(9)<br>*MAG*(8)<br>*HJURP*(7)<br>*TMEM125*(7) |
| 0.95 | 0.01 | 61 | 74 | 66 | 65 | 13 | 12 | *KIFC1*(10)<br>*TOP2A*(10)<br>*TMEM125*(7)<br>*MAG*(6)<br>*UBE2C*(6) | *KIFC1*(10)<br>*MAG*(7)<br>*TMEM125*(7)<br>*TOP2A*(7)<br>*UBE2C*(6) |
| 0.97 | 0.001 | 12 | 6 | 4 | 3 | 6 | 3 | *ANXA2P2*(1)<br>*ANXA2*(1)<br>*C1QB*(1)<br>*C1QC*(1)<br>*COL4A1*(1) | *KLK6*(2)<br>*C1QB*(1)<br>*C1QC*(1)<br>*GJB1*(1)<br>*MAG*(1) |
| 0.97 | 0.005 | 9 | 5 | 4 | 4 | 4 | 3 | *KLK6*(2)<br>*ANXA2P2*(1)<br>*ANXA2*(1)<br>*C1QB*(1)<br>*C1QC*(1) | *KLK6*(2)<br>*C1QB*(1)<br>*C1QC*(1)<br>*GJB1*(1)<br>*MAG*(1) |
| 0.97 | 0.01 | 7 | 4 | 4 | 4 | 3 | 3 | *KLK6*(2)<br>*C1QB*(1)<br>*C1QC*(1)<br>*GJB1*(1)<br>*MAG*(1) | *KLK6*(2)<br>*C1QB*(1)<br>*C1QC*(1)<br>*GJB1*(1)<br>*MAG*(1) |

Table S3: JGL parameter impact in case (C) astrocytoma vs. oligoddendroglioma vs. GBM (glioblastoma).

| $\lambda_1$ | $\lambda_2$ | Connected Nodes | Astrocytoma Edges | Oligodendroglioma Edges | GBM Edges | Shared Edges by 3 classes | Astrocytoma Subnetworks | Oligodendroglioma Subnetworks | GBM Subnetworks | 5 Hubs Astrocytoma | 5 Hubs Oligoendroglioma | 5 Hubs GBM |
|---|---|---|---|---|---|---|---|---|---|---|---|---|
| 0.9 | 0.001 | 689 | 2270 | 2028 | 538 | 179 | 56 | 51 | 67 | *NCAPG*(64)<br>*BUB1B*(62)<br>*TPX2*(61)<br>*FAM64A*(59)<br>*CDK1*(58) | *HJURP*(49)<br>*MELK*(47)<br>*VSNL1*(47)<br>*TOP2A*(46)<br>*KIFC1*(44) | *CD53*(25)<br>*VAV1*(25)<br>*LAPTM5*(24)<br>*NCKAP1L*(24)<br>*SPI1*(24) |
| 0.9 | 0.005 | 543 | 1874 | 1674 | 462 | 253 | 45 | 47 | 59 | *NCAPG*(59)<br>*BUB1B*(56)<br>*KIFC1*(53)<br>*FAM64A*(52)<br>*NCAPH*(52) | *MELK*(46)<br>*HJURP*(45)<br>*NCAPG*(45)<br>*TOP2A*(45)<br>*KIFC1*(43) | *CD53*(25)<br>*LAPTM5*(24)<br>*NCKAP1L*(23)<br>*SPI1*(23)<br>*VAV1*(23) |
| 0.9 | 0.01 | 428 | 1489 | 1348 | 410 | 321 | 43 | 47 | 50 | *NCAPG*(49)<br>*FAM64A*(48)<br>*NUSAP1*(48)<br>*TOP2A*(47)<br>*TPX2*(47) | *HJURP*(42)<br>*KIFC1*(42)<br>*TOP2A*(42)<br>*NCAPG*(41)<br>*MELK*(40) | *LAPTM5*(22)<br>*VAV1*(20)<br>*NCKAP1L*(19)<br>*CD53*(18)<br>*SPI1*(18) |
| 0.95 | 0.001 | 95 | 108 | 73 | 23 | 9 | 15 | 10 | 14 | *TOP2A*(13)<br>*NUSAP1*(11)<br>*FAM64A*(10)<br>*KIFC1*(9)<br>*TPX2*(9) | *KIFC1*(9)<br>*MAG*(8)<br>*TOP2A*(8)<br>*UBE2C*(6)<br>*CARNS1*(5) | *KLK6*(3)<br>*MAG*(3)<br>*ANXA2P1*(2)<br>*ANXA2P2*(2)<br>*ANXA2*(2) |
| 0.95 | 0.005 | 55 | 49 | 42 | 20 | 13 | 11 | 9 | 12 | *MAG*(6)<br>*TOP2A*(6)<br>*TMEM125*(5)<br>*CARNS1*(4)<br>*FAM64A*(4) | *MAG*(7)<br>*TMEM125*(5)<br>*CARNS1*(4)<br>*FAM64A*(4)<br>*GJB1*(4) | *MAG*(3)<br>*ANXA2P1*(2)<br>*ANXA2P2*(2)<br>*ANXA2*(2)<br>*C1QA*(2) |
| 0.95 | 0.01 | 38 | 29 | 28 | 17 | 15 | 11 | 10 | 10 | *MAG*(6)<br>*GJB1*(3)<br>*KLK6*(3)<br>*TMEM125*(3)<br>*TOP2A*(3) | *MAG*(6)<br>*GJB1*(3)<br>*KLK6*(3)<br>*TMEM125*(3)<br>*TOP2A*(3) | *MAG*(4)<br>*C1QA*(2)<br>*C1QB*(2)<br>*C1QC*(2)<br>*DOCK2*(2) |
| 0.97 | 0.001 | 11 | 4 | 4 | 3 | 1 | 4 | 3 | 3 | *ANXA2P2*(1)<br>*ANXA2*(1)<br>*C1QB*(1)<br>*C1QC*(1)<br>*GJB1*(1) | *KLK6*(2)<br>*C1QB*(1)<br>*C1QC*(1)<br>*GJB1*(1)<br>*MAG*(1) | *ANXA2P2*(1)<br>*ANXA2*(1)<br>*C1QB*(1)<br>*C1QC*(1)<br>*RPSAP58*(1) |
| 0.97 | 0.005 | 4 | 2 | 1 | 2 | 1 | 2 | 1 | 2 | *ANXA2P2*(1)<br>*ANXA2*(1)<br>*C1QB*(1)<br>*C1QC*(1) | *C1QB*(1)<br>*C1QC*(1) | *ANXA2P2*(1)<br>*ANXA2*(1)<br>*C1QB*(1)<br>*C1QC*(1) |
| 0.97 | 0.01 | 2 | 1 | 1 | 1 | 1 | 1 | 1 | 1 | *C1QB*(1)<br>*C1QC*(1) | *C1QB*(1)<br>*C1QC*(1) | *C1QB*(1)<br>*C1QC*(1) |

## RSKC complete results and supplementary validation figures

The complete results of RSKC are reported in Table S4. The used trimming parameter, $\alpha$, was set to 0.1. The values of penalization, $L1$, and the number of clusters, $K$,

Table S4: Summarized results of applying sparse clustering method RSKC to the complete dataset and our defined cases. In addition to the values of the tuning parameters $K$ and $L1$, the number of selected features in each case is reported ('No. Selected' column). 'Complete dataset' refers to the original dataset.Cases A, B and C represent our case of study, respectively, 'LGG vs GBM', 'Astrocytoma vs Oligodendroglioma', and 'Astrocytoma vs Oligodendroglioma vs GBM', and the corresponding dataset is constituted by the genes selected by JGL method (with $\lambda_1 = 0.095$ and $\lambda_2 = 0.01$). For each dataset, the number of starting variables is reported in squared brackets. *Silhouette* and *Calinski-Harabasz* columns show the values of the two scores used to evaluate clustering performances, computed by considering all samples, and by excluding the outliers (the values in brackets). ARI was calculated in the most representative cases, to quantify the accordance between the known labels and the identified clusters. LGG: Lower-Grade Glioma; GBM: glioblastoma.

| Dataset [number of variables] | K | L1 | No. Selected | $Silhouette$ | $Calinski-Harabasz$ | $ARI$ |
|---|---|---|---|---|---|---|
| Complete dataset [163338] | 2 | 2 | 6 | 0.82 (0.86) | 1367.10 (1486.03) | 0.55 |
| | 2 | 24 | 967 | 0.60 (0.64) | 434.15 (442.44) | – |
| | 3 | 2 | 5 | 0.57 (0.58) | 443.30 (498.74) | $5.5 \cdot 10^{-3}$ |
| | 3 | 24 | 828 | 0.37 (0.41) | 263.49 (268.72) | – |
| Case (A) LGG vs GBM [43] | 2 | 2 | 6 | 0.70 (0.74) | 640.95 (715.47) | 0.54 |
| | 2 | 24 | 43 | 0.45 (0.48) | 172.03 (182.28) | – |
| | 3 | 2 | 7 | 0.62 (0.65) | 746.37 (785.49) | – |
| | 3 | 24 | 43 | 0.41 (0.47) | 165.04 (177.61) | – |
| Case (B) Astrocytoma vs Oligodendroglioma [61] | 2 | 2 | 6 | 0.57 (0.64) | 513.29 (579.03) | 0.15 |
| | 2 | 24 | 61 | 0.25 (0.30) | 72.58 (80.33) | – |
| | 3 | 2 | 7 | 0.59 (0.61) | 526.92 (558.90) | |
| | 3 | 24 | 61 | 0.28 (0.31) | 75.54 (77.47) | – |
| Case (C) Astrocytoma vs Oligodendroglioma vs GBM [107] | 2 | 2 | 6 | 0.75 (0.77) | 843.31 (907.61) | 0.49 |
| | 2 | 24 | 38 | 0.42 (0.48) | 172.13 (185.03) | – |
| | 3 | 2 | 6 | 0.61 (0.65) | 753.07 (816.78) | 0.21 |
| | 3 | 24 | 38 | 0.41 (0.48) | 170.33 (185.79) | – |

are shown in the table. '$Silhouette$' and '$Calinski - Harabasz$' are the calculated scores. The three cases of study were compared with the value obtained by starting from the complete dataset. The number of variables in the considered dataset is reported in squared brackets the table. Despite the considerable variable reduction, the *silhouette* and *Calinki-Harabasz* values of the cases of study are satisfactory, also in comparison with the one provided by the complete dataset.

As a result of the performed clustering validation, we obtain an estimate of the distribution of both *silhouette* and *Calinski-Harabasz* scores over the 1000 random sets. Figures S1 and S2 show the histograms of the two obtained score distributions for the different parameter combinations. In each chart, the red arrow indicates the value of the score obtained by considering the set of selected variables (reference score). In all cases, for both $K = 2$ and $K = 3$ the scores are either better than the one computed for the best random set, or in the upper quantile (as it occurs in case B for $K = 2$).

images/histK2.png

Figure S1: Distribution of silhouette and Calinski-Harabasz scores for clustering derived from 1000 random datasets ($K = 2$). On x-axis the values of the two indexes are reported. Y-axis shows the frequency. Red arrows highlight the score computed by considering our set of selected variables. When the corresponding value is not available (too high than the best computed score of random selection) the arrow points to the right. Cases A, B and C represent our case of study 'LGG vs GBM', 'Astrocytoma vs Oligodendroglioma', and 'Astrocytoma vs Oligodendroglioma vs GBM', respectively.

images/histK2.png

Figure S2: Distribution of silhouette and Calinski-Harabasz scores for clustering derived from 1000 random datasets ($K = 3$). On x-axis the values of the two indexes are reported. Y-axis shows the frequency. Red arrows highlight the score computed by considering our set of selected variables. When the corresponding value is not available (too high than the best computed score of random selection) the arrow points to the right. Cases A, B and C represent our case of study 'LGG vs GBM', 'Astrocytoma vs Oligodendroglioma', and 'Astrocytoma vs Oligodendroglioma vs GBM', respectively.

## 0.2 Literature research

In Table S5, we summarize the main knowledge discovered by our literature research. For each gene (or group of genes), the main knowledge and the corresponding references are reported. Genes that are either jointly studied, or identified based on their estimated relations, are discussed together.

Table S5: Literature research outcome.

| **Genes** | **Knowledge** | **Reference** |
|---|---|---|
| *PBK*<br>*FAM64A*<br>*KIFC1* | • *PBK*, also known as *TOPK*, is a protein-coding genes. The corresponding protein is overexpressed in various cancers, such as gastric adenocarcinoma and breast cancer. It is deferentially expressed in the glioma types, where low expression is typical of low grades, while high-grades are associated to high expression. It has been recently proposed as prognostic biomarker in GBM.<br>• *FAM64A* encourage carcinogenesis by its differential expression in a variety of cancers. In glioma, high-expression is associated with poor prognosis.<br>• *KIFC1* is involved in cell division, and it is essential for cancer growth, since it allows the survival of cells with abnormal centrosome number, as it occurs in cancer. It is indicated as target for several cancers, such as hepatocellular carcinoma and prostate cancer, and it has been recently investigated for its role in Temozolomide resistance in glioma. | [78, 79, 56, 80, 81, 57, 82, 83, 84, 69] |
| *UBE2C*<br>*AURKB* | These two genes have been studied in cancer for their contribution in cell division and other cell regulatory processes. *UBE2C* expression has been associated with malignant glioma for many years. Recently, it has been discovered that the combined expression of *AURKB* and *UBE2C* is linked with histological features (the basis of 2016 glioma classification). In particular, in high-grade gliomas these genes are co-overexpressed, while in low-grade gliomas are poorly expressed. | [85, 86, 70, 59, 60] |
| *C1QA*<br>*C1QB*<br>*C1QC* | A bioinformatic study compared the expression levels of *C1QA*, *C1QB*, and *C1QC* genes, discovering a prevalence of high expression in glioma cells compared to normal brain tissue. Survival analysis on transcriptomics data revealed an association between the gene expression and poor prognosis. | [87] |
| *ANXA2* | This gene is involved in many cancer mechanisms, such as cell proliferation, migration, angiogenesis, metastasis and therapy resistance. Bioinformatic studies revealed *ANXA2* overexpression in several cancers, including glioma, and a positive correlation with glioma aggressiveness. Survival analysis associates this gene with patient' survival. | [88, 89, 68] |
| *CENPF* | It is involved in several cancer-related functions. Transcriptomics analysis based on multiple databases identified *CENPF* as a potential biomarker for glioma. | [72] |

| | | |
|---|---|---|
| *NCAPH* | A bioinformatics study found up-regulation of *NCAPH* in high-grade glioma tissues. Survival analysis revealed the association between *NCAPH* and poor glioma prognosis. | [73] |
| *ERMN* *MOBP* | Both genes code for myelin-related proteins involved in oligodendrocytes differentiation A gene-coexpression network analysis revealed both *ERMN* and *MOBP* as up-regulated in GBM tissues, and identified them as specific GBM biomarkers. | [90, 91, 74] |
| *CARNS1* | This gene has been investigated in breast cancer through bioinformatic analysis, which revealed *CARNS1* as a potential prognostic marker. In particular, *CARNS1* acts as tumor-suppressor gene, which is silenced in breast cancer. | [76] |
| *DOCK2* | This gene is associated with the proliferation of cancer cells and plays a role in cell motility. Even if *DOCK2* has not been studied in the context of adult-glioma, it has been identified as promising therapeutic target for the treatment of pediatric astrocytoma. | [92, 93] |
